# Chemotherapy induced juvenile dermatomyositis: a novel presentation- a case report

**DOI:** 10.1186/s12887-022-03704-5

**Published:** 2022-10-31

**Authors:** Keta Vagha, Chitturi Venkata Sai Akhil, Spandana Madirala, Ashish Varma, Mahaveer Lakra, Jayant Vagha

**Affiliations:** Department of Pediatrics, Jawaharlal Nehru Medical College, Acharya Vinoba Bhave Rural Hospital, Sawangi Meghe, Wardha, Maharashtra India

**Keywords:** Dermatomyositis, Chemotherapy, Malignancy, Inflammatory myositis

## Abstract

**Background:**

Idiopathic connective tissue disease juvenile dermatomyositis (JDM) is characterised by inflammatory myositis and distinctive skin abnormalities. Only a few cases of Dermatomyositis (DM) owing to chemotherapy used to treat cancer have been reported, despite the fact that the link between DM and cancer in adults is widely known. We describe the case of a female, age 14, who experienced DM as a side effect of chemotherapy following enucleation for retinoblastoma. We also discussed our patient's likely pathophysiology of JDM after treatment.

**Case presentation:**

A 14-year-old female came to our facility complaining of trouble walking and bluish-black discoloration on her neck, elbows, forehead, and knees that had been present for eight months. The patient had undergone enucleation of the left eye due to retinoblastoma, followed by 40 cycles of radiation therapy and 13 cycles of chemotherapy with Cyclophosphamide, Etoposide, Carboplatin, Vincristine, and Dactinomycin. Her serum LDH and CPK levels were high, and she tested positive for ANA. The muscle biopsy was consistent with the changes of DM. When electromyography was performed, it revealed tiny, fibrillating, polyphasic motor unit potentials and sharp, positive waves that were suggestive with DM. A diagnosis of JDM was made after taking into account the symptoms, biochemical data, muscle biopsy, and electromyography results. The patient's symptoms started to get better once methotrexate and oral corticosteroids were started.

**Conclusion:**

This case report emphasises the value of ongoing observation after cancer chemotherapy because specific cutaneous and muscle symptoms may lead paediatricians to consider the possibility of chemotherapy-induced JDM, which is uncommon in young patients.

## Introduction

DM is a type of idiopathic inflammatory myopathy that manifests as gradual, symmetrical weakening in the proximal muscles as well as characteristic cutaneous changes [1]. It could involve different organs like the heart, stomach, intestines, lungs, etc. Despite being uncommon, DM is the most common form of idiopathic inflammatory myopathy. According to reports in the adult population, 15 to 30 percent of DM patients have an underlying malignancy, and these individuals have a 5–7 times higher risk of getting cancer than the general population [2]. However, there is a dearth of such information regarding children. Post-chemotherapy DM is a rare occurrence, especially in kids. Here, we describe a rare instance of JDM that manifested in a girl aged 14 after undergoing 40 cycles of radiotherapy and 13 cycles of chemotherapy following enucleation for retinoblastoma. The title of our case, "Chemotherapy caused Juvenile Dermatomyositis," so distinguishes it from others.

## Case presentation

A 14-year-old female came to our facility with concerns about difficulty in walking owing to weakness in both lower limbs, an erythematous, pruritic rash that covered her face, neck, axilla, elbows, and knees, and photosensitivity that had been present for eight months. It was unknown if she had any allergies. Patient underwent surgery 1.5 years after being diagnosed with retinoblastoma two years prior. After the enucleation, the patient underwent 40 cycles of radiation therapy and 13 cycles of chemotherapy with the drugs cyclophosphamide, etoposide, carboplatin, vincristine, and dactinomycin. In addition to developing proximal muscular weakness in both the upper and lower body after undergoing chemotherapy for 10 months, the patient also got a rash over their face that progressed.

**Fig. 1:**
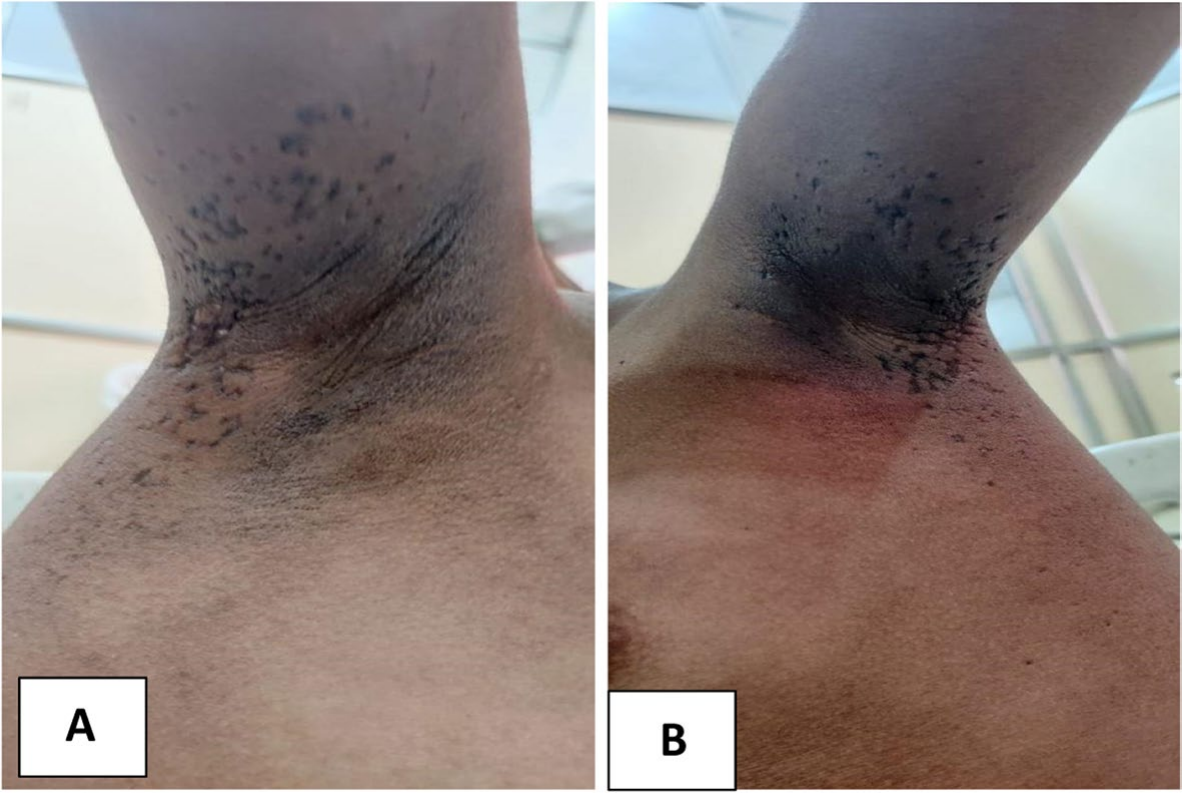
**A** and **B** show the lichenified plaques in the right and left axilla

**Fig. 2:**
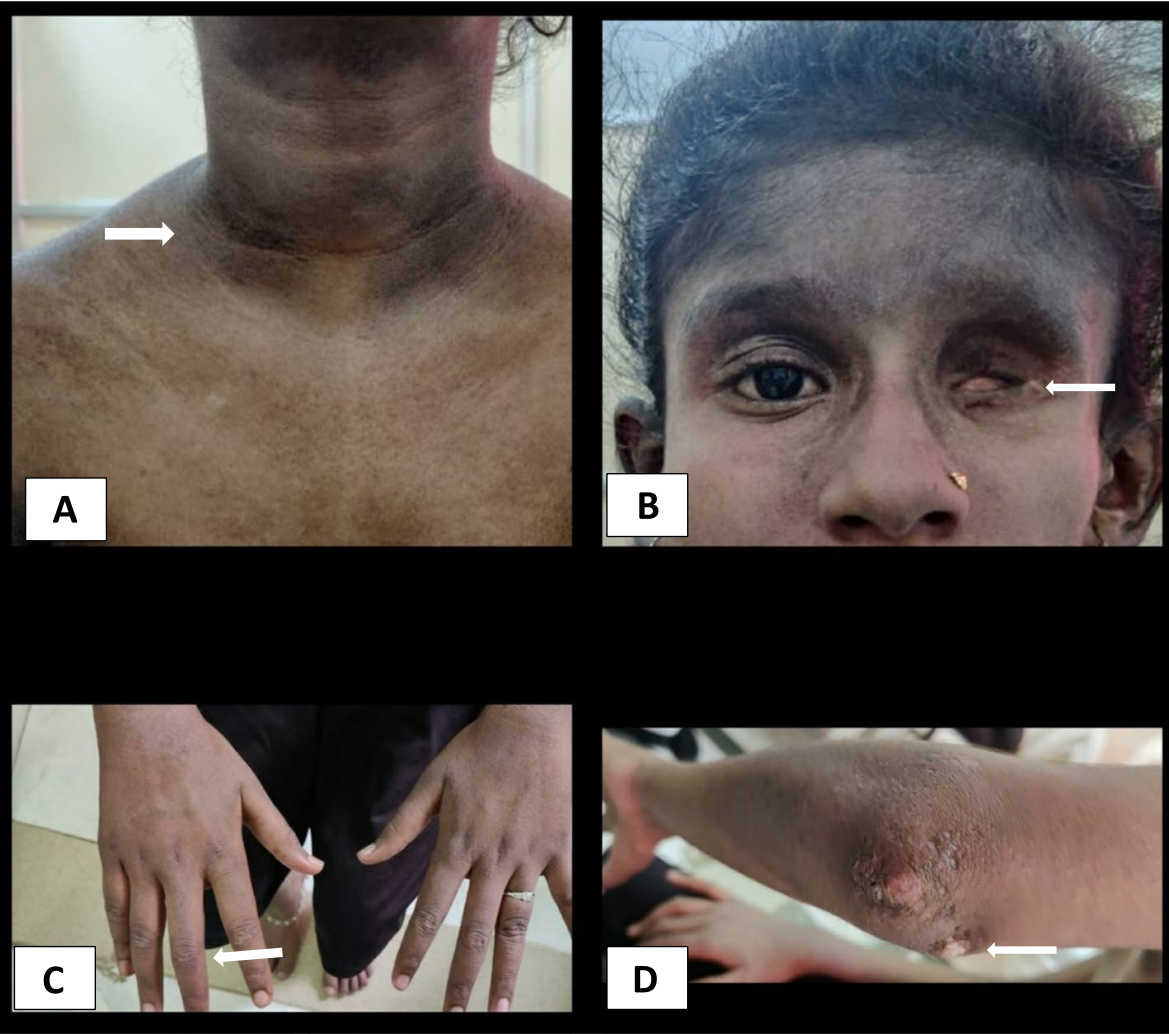
**A** The V neck sign/Shawl sign on the neck and upper chest. **B** Heliotrope rash with erythema over the face, Arrow represents the site of enucleation. **C** Gottron’s papules over the digits which are in the healing stage. **D** Pearly white lesion on the extensor aspect of the elbow showing Calcinosis cutis

The patient was brought with these issues to our facility. Indicators of the patient's weakness in the proximal muscles of the upper and lower limbs were difficulty in rising from a sitting position and lifting the arms over the head. Her lower limbs had grade II muscle power and her upper limbs grade III, according to the examination. The CMAS score for childhood myositis was 36. Her vital signs were normal, and she had no underlying systemic issues. On inspection of the joints, there were no indications of active synovitis. At presentation, her neck flexor strength was higher than grade IV. Findings of skin examination showed hallmark manifestations consistent with DM, well defined to ill defined hyperpigmented scaly lichenified plaques over the neck, face, axilla (Fig. 1A, 1B), elbows, knees, including the V neck sign/Shawl sign(Fig. 2A), heliotrope eruption over the face especially on the forehead and periorbital region (Fig. 2B), Gottron papules over the digits of upper limb on both sides (Fig. 2C), calcinosis cutis involving the extensor aspect of elbows (Fig. 2D).

**Fig. 3:**
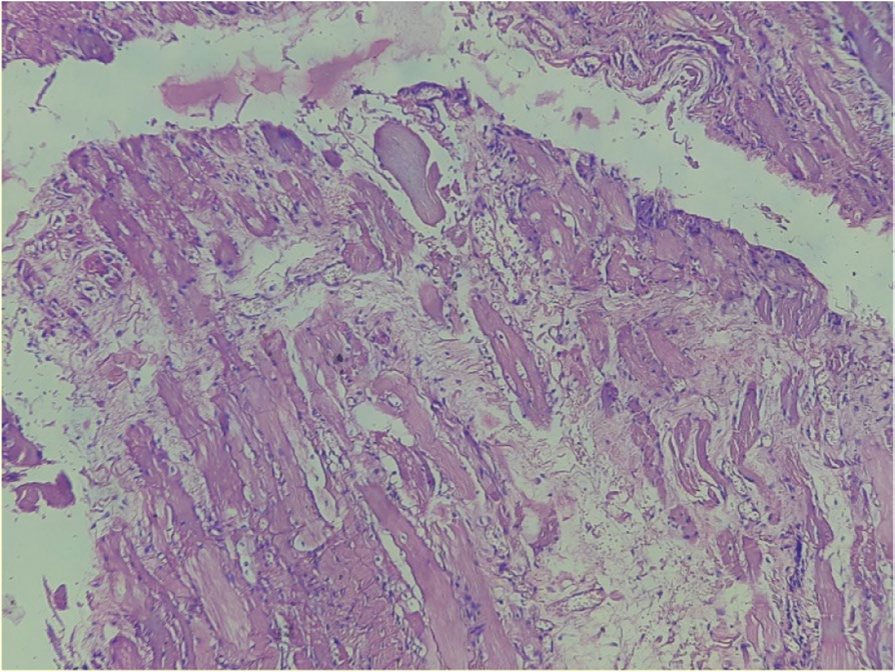
Given section stained with Hematoxylin and Eosin (low power view: 10x) shows fibroadipose tissue, blood vessels and chronic inflammatory response around the blood vessels (perivascular). Muscle fibres in the dermis appear necrosed and fragmented

The initial blood tests, such as complete blood counts, liver and renal functions, were all normal. Both lactate dehydrogenase (578 U/L) and creatine phosphokinase (171.61 U/L) levels were high. Positive antinuclear antibodies were found. Features of Dermatomyositis with interface dermatitis and mucin deposition in the dermis was consistent in the skin punch biopsy. Electromyography was done which showed small polyphasic motor unit potentials with fibrillations, positive sharp waves suggestive of Dermatomyositis. A muscle biopsy revealed fibres of the muscle that were necrosed and fragemted [Fig. 3].

**Fig. 4:**
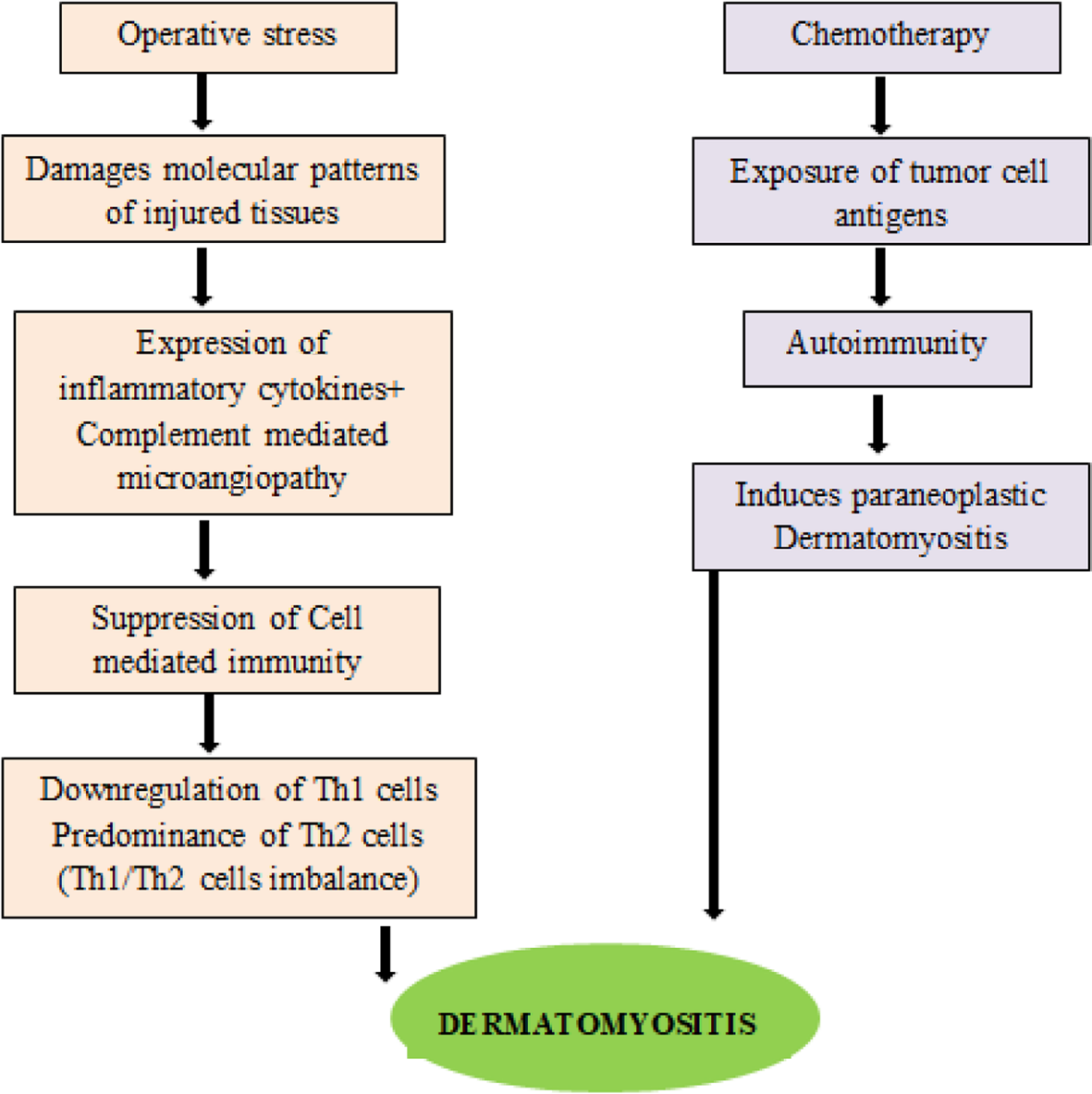
Potential pathogenesis of malignancy associated DM

**Fig. 5:**
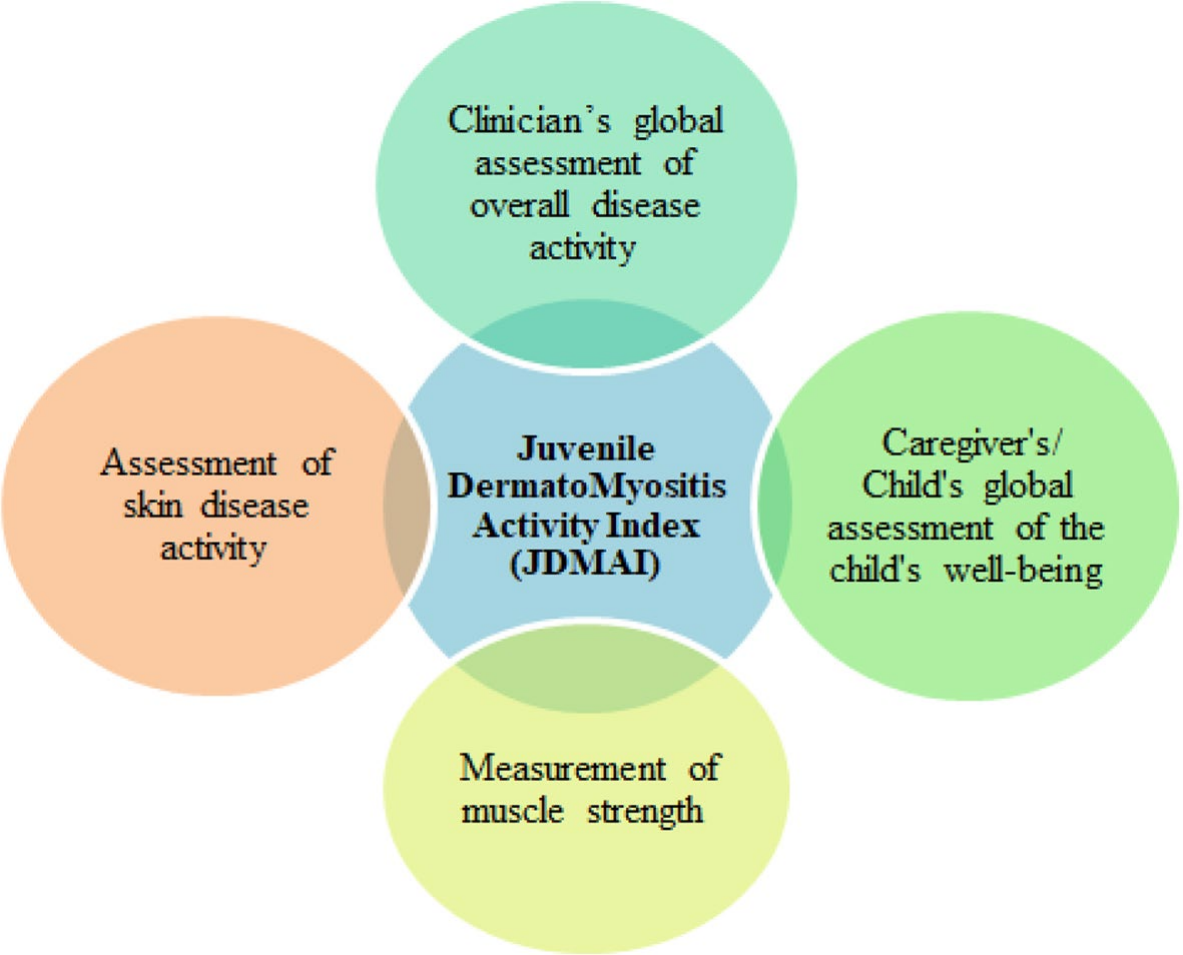
Evaluation of Response to therapy in JDM

According to the online calculator of EULAR/ACR classification for Juvenile Idiopathic Inflammatory Myopathies(IIM), the criterion score was 15.7, which was suggestive of Definite IIM [3]. According to the Bohan and Peter criteria [4], JDM was determined to be the cause of the symptoms, which included the characteristic heliotrope rash with Gottron papules, symmetrical proximal weakness, raised muscle enzyme levels, electromyographic alterations, and even JDM-related changes in the muscle biopsy.

Prednisolone oral 2 mg/kg/day and methotrexate oral 15 mg/m2 once weekly were started on the patient. After beginning the therapy for a week, the youngster displayed a slight improvement in symptoms as shown by lessened joint discomfort, stiffness, and erythema. Exercises for muscle strengthening and physiotherapy were started. During the patient's future hospital visits, it was seen that the patient's muscle strength had significantly improved.

## Discussion

DM is a female-predominant idiopathic autoimmune connective tissue disease that is frequently accompanied by proximal muscle weakness and skin rashes [5]. The degree of cutaneous and muscular symptoms varies, and DM can be categorised as:

- Classic DM: It is characterised by simultaneous cutaneous and muscle involvement [6].
- Amyopathic DM: Despite having cutaneous indications that are consistent with DM for at least six months, these individuals lack muscular weakness and show no laboratory or radiologic evidence of myositis [6].
- Hypomyopathic DM: Muscle enzyme serologic tests, electromyography, muscle biopsy, or magnetic resonance imaging all show subclinical signs of myositis (MRI) [6].
- Postmyopathic DM: When muscle illness is treated with immunosuppressive medication, cutaneous symptoms continue to exist [6].

Obtaining a thorough history and doing a physical examination are part of the first workup for DM cases. The severity of muscle weakness does not always correspond to the degree of CK increase [7]. Elevated levels of lactate dehydrogenase, aspartate aminotransferase, and alanine aminotransferase can also be seen, indicating muscular involvement. Nearly 60% of patients have negative antinuclear antibody levels [7]. Up to 80% of cases have myositis-specific antibodies, which can help define a serologic profile and indicate extramuscular symptoms and prognosis [8]. Our case report had a limitation that we were unable to test the myositis-specific antibodies (MSAs) and myositis-associated antibodies (MAAs), which prevented us from being clear about the subgroup of DM. For use in clinical and research settings, the EULAR/ACR classification criteria for Juvenile IIM provide a patient's likelihood of having IIM [3]. The likelihood is determined by adding the weights assigned to a group of criteria elements, which results in a score.

Numerous malignancies, including breast, ovarian, lung, and hematologic cancers as well as nasopharyngeal carcinoma in Asian populations, have been linked to DM. When DM is diagnosed, a thorough check for cancer is crucial. Here, we present a rare instance of DM brought on by post-enucleation chemotherapy and radiation treatment for retinoblastoma. Our case report has special significance because it is rather rare for DM to occur after a cancer diagnosis and chemotherapy.

Operative stress and chemotherapy are two suggested pathogenic potentials (Fig. 4) [9]. JDM may potentially result from a Th1/Th2 immune system imbalance, according to a theory [10]. Chemotherapy may have played a significant role in the development of JDM in our case because the cutaneous signs of JDM only emerged after the start of chemotherapy. In general, chemotherapy suppresses the immune system and treats autoimmune diseases. There have been reports that chemotherapy exposes tumour cell antigens and triggers autoimmune [10]. Since the patient had undergone surgery long before developing complaints, we considered chemotherapy for the development of DM in our report rather than operative stress. The patient's skin manifestations didn't appear until after chemotherapy had started and before all of the cycles had been completed.

A 53-year-old man with enlarged cervical lymph nodes was described by Yu Fujiwara et al. (Japan, 2020), and a biopsy revealed embryonal cancer in this case. After one month of treatment with the Bleomycin, Etoposide, and Cisplatin (BEP) regimen, the patient had developed skin lesions of DM [11]. Yuki Otsuka et al. (Japan, 2017) described a case of a 61-year-old woman who had invasive ductal breast cancer and had developed DM [12]. Yuta Ito et al. (Japan, 2017) described a case of an ovarian cancer patient, age 50, who acquired diabetes after undergoing hysterectomy, adnexectomy, and chemotherapy with Paclitaxel and Carboplatin (PTX + CBDCA) [13]. They had hypothesised that cytokines such tumour necrosis factor (TNF)- may be crucial in the development of scleroderma/DM caused by taxanes. After receiving chemotherapy with Carboplatin and Capecitabine, a 76-year-old man with a recent diagnosis of metastatic gastric adenocarcinoma developed diabetes mellitus, according to a case study by Xiaolong et al. (UK, 2014) [14]. A few of the many medications that have a higher degree of correlation with DM are etoposide, carboplatin, paclitaxel, bleomycin, cisplatin and cyclophosphamide. We attribute the development of DM to chemotherapy rather than the malignancy of Retinoblastoma because there is no evidence linking it to the development of DM. Our patient received etoposide and carboplatin for 13 cycles during which time she developed the hall cutaneous manifestations of DM.

Control of the underlying inflammatory myositis and the avoidance or treatment of complications are the objectives of treatment (Contractures and Calcinosis) [15]. Photoprotection, anti-pruritic medications, topical corticosteroids or calcineurin inhibitors, systemic corticosteroids, or methotrexate are all parts of a multifaceted strategy to treating JDM [15]. The first line of treatment for children with mild to severe JDM is a combination of high-dose oral prednisone (2 mg/kg per day) and methotrexate (15 mg/m2, once weekly subcutaneous injection) [16]. The cumulative dose of corticosteroids and their negative side effects are decreased by this combination medication [17]. Patients on oral Prednisone therapy may experience a poor response or worsening of their condition; in these cases, intravenous methylprednisolone (IVMP) or intravenous immunoglobulin is added.

The Juvenile Dermatomyositis Activity Index (JDMAI) used a composite score made up of four components to make the evaluation of treatment response simpler (Fig. 5) [18].

Over the course of 6 to 8 weeks of therapy, the effectiveness of the treatment is clinically observed for improved muscle strength and cutaneous symptom alleviation. Typically, a decrease in Creatine kinase levels is first observed throughout the healing process, followed by a more gradual return of muscle strength. The prednisone dose can be reduced once clinical improvement is seen [18]. In connecting the patient's history with clinical indicators, our case study emphasises the importance of clinical judgement. A multidisciplinary strategy combining family doctors, rheumatologists, dermatologists, and medical oncologists is crucial for the management of JDM.

## Conclusion

To the best of our knowledge, chemotherapy-induced juvenile dermatomyositis is a rare occurrence, in contrast to a number of earlier findings that focused on the temporal relationship between dermatomyositis and malignancy. A delayed diagnosis of DM would be detrimental to the patient's survival and quality of life.

## Data Availability

The datasets analysed during the current study was available from the corresponding author on reasonable request.

## Abbreviations

JDM: Juvenile Dermatomyositis
ANA: Anti-nuclear antibodies
LDH: Lactate Dehydrogenase
CPK: Creatine Phosphokinase
DM: Dermatomyositis
EMG: Electromyography
MRI: Magnetic Resonance Imaging
CK: Creatine Kinase
IVMP: Intravenous Methylprednisolone
JDMAI: Juvenile Dermatomyositis Activity Index
